# Using the Health Belief Model to Understand Why Making Oral HIV Self-Testing Available to Truck Drivers in Kenya Had Little Impact on Six-Month Testing

**DOI:** 10.1007/s10461-024-04500-1

**Published:** 2024-10-19

**Authors:** Thae Aient Aient Oo, Matthew L. Romo, Gavin George, Eva Mwai, Eston Nyaga, Joanne E. Mantell, Jacob O. Odhiambo, Kaymarlin Govender, Elizabeth A. Kelvin

**Affiliations:** 1 Department of Epidemiology and Biostatistics, CUNY Graduate School of Public Health and Health Policy, New York, NY 10027 USA; 2 CUNY Institute for Implementation Science in Population Health, City University of New York, New York, NY USA; 3 Health Economics and HIV and AIDS Research Division, University of KwaZulu-Natal, Durban, South Africa; 4 Division of Social Medicine and Global Health, Lund University, Lund, 22363 Sweden; 5 North Star Alliance, Nairobi, Kenya; 6 HIV Center for Clinical and Behavioral Studies, Department of Psychiatry, Gender, Sexuality and Health Area, New York State Psychiatric Institute and Columbia University Irving Medical Center, New York, NY USA; 7 Department of Occupational Health, Epidemiology & Prevention, Donald and Barbara Zucker School of Medicine, Hofstra University/Northwell Health, Hempstead, NY USA

**Keywords:** HIV, Self-testing, Kenya, Truck drivers, Health Belief Model, Fatalism, Self-efficacy, Risk pereption

## Abstract

Research has found that offering HIV self-testing (HIVST) to truckers in Kenya increased testing rates at baseline but not over 6-month follow-up. We explored possible explanations based on the Health Belief Model by assessing HIV risk perception, self-efficacy, and fatalism as possible effect modifiers of the impact of offering HIVST (intervention *n* = 150) versus standard of care (SOC *n* = 155) on 6-month testing on the multiplicative and additive scales using log binomial and linear binomial regression and stratifying on significant modifiers. We found significant interaction between the intervention and fatalism on both the multiplicative (*p* = 0.020) and additive (*p* = 0.020) scales. In the stratified models, the HIVST intervention was associated with higher HIV testing among participants with low fatalism but lower testing among those with high fatalism (risk ratio [RR] = 1.30, *p* = 0.065 versus RR = 0.74, *p* = 0.072; risk difference [RD] per 100 = 14.00, *p* = 0.080 versus RD=-14.69, *p* = 0.086). Truckers in Kenya are described as being highly fatalistic, feeling lack of control over their lives and health. We found that fatalistic views negated the potential benefit of offering HIVST to truckers. For HIVST to have an impact among truckers, psychosocial interventions may be needed that address fatalistic views.

## Resumen

Investigaciones han encontrado que ofrecer la autoprueba del VIH (AP-VIH) a los camioneros en Kenia aumentó las tasas de pruebas al inicio pero no durante el seguimiento de 6 meses. Hemos explorado posibles explicaciones basadas en el Modelo de Creencias de Salud, evaluando la percepción de riesgo para el VIH, la autoeficacia, y el fatalismo como posibles modificadores del impacto de ofrecer la AP-VIH (intervención *n* = 150) versus atención estándar (AE *n* = 155) en la probabilidad de hacer la prueba del VIH durante 6 meses de seguimiento en la escala multiplicativa y aditiva utilizando regresión log binomial y linear binomial y estratificación en modificadores significativos. Encontramos una interacción significativa entre la intervención y el fatalismo en la escala multiplicativa (*p* = 0,020) y aditiva (*p* = 0,020). En los modelos estratificados, la intervención AP-VIH se asoció con mayor índice de prueba de VIH entre participantes con bajo fatalismo, pero con menor índice de prueba entre aquellos con alto fatalismo (riesgo relativo [RR] = 1,30, *p* = 0,065 versus RR = 0,74, *p* = 0,072; diferencia de riesgo [DR] por 100 = 14.00, *p* = 0.080 versus DR=-14.69, *p* = 0.086). Los camioneros en Kenia son descritos como muy fatalistas y sienten falta de control sobre sus vidas y su salud. Encontramos que las opiniones fatalistas niegan el beneficio potencial de ofrecer la AP-VIH entre los camioneros. Para que la AP-VIH tenga un impacto positivo entre los camioneros, intervenciones psicosociales pueden ser necesarias para abordar las opiniones fatalistas.

## Introduction

Truck drivers are essential workers [1] and ensuring their access to and uptake of preventive healthcare is key to maintain supply chains. Truckers were severely impacted during the early stages of the HIV epidemic [2] and continue to be at high HIV risk due to their mobility and multiple sexual partnerships along their trucking route [3]. From 1993 to 2011, HIV prevalence among truck drivers was between 15% and 56% in various regions of Africa, and around 26% in Kenya [4]. A study among truck drivers in Kenya found that a high proportion reported transactional sex (56%) and regular partnerships along their trucking route in addition to having a wife or girlfriend at home (47%) [5]. Another study found that compared to the general population, Kenyan truck drivers were less likely to use condoms with both regular and casual partners [6]. Given this high-risk sexual behavior, the Kenyan National AIDS and STI Control Program (NASCOP) designated truck drivers as a high-risk population to monitor in 2014 [7].

Screening for HIV is a key step in achieving the UNAIDS 95-95-95 fast-track targets (95% of people living with HIV know their status, 95% of those diagnosed with HIV are on treatment, and 95% of those on treatment are virally suppressed) [8] and keeping people healthy and working. A systematic review regarding HIV self-test (HIVST) uptake among men in sub-Saharan Africa found that HIVST was considered more convenient than traditional clinic-based testing [9]. Access to clinic-based healthcare services is a challenge for truckers due to work-related travel and irregular schedules [10], and HIVST may be easier to access. Numerous randomized controlled trials (RCT) have found that offering HIVST increases HIV testing [11–24]. We conducted a RCT evaluating the impact of adding HIVST as an option for truckers through a roadside clinic system in Kenya. Similar to other studies, at baseline we found that those offered a choice between the standard of care (SOC) provider-administered finger-prick rapid HIV testing or a rapid oral HIVST were more likely to test compared to those only offered SOC testing [5]. However, we found no impact of making the HIVST kits available through this clinic system over a 6-month follow-up period [25].

The Health Belief Model (HBM) [26, 27] suggests that uptake of preventive health behaviors, such as HIV testing, is influenced by six factors: (a) perceived severity, (b) perceived barriers, (c) cue to action, (d) perceived susceptibility, (e) self-efficacy, and (f) perceived benefits. HIVST might increase HIV testing rates by addressing perceived barriers (e.g., time it takes to test in a clinic, concerns over confidentiality, or concerns over being able to handle an HIV-positive test result away from family) (HBM factor b) and serving as a cue to action (i.e., being told about and offered something novel may encourage people to try it, at least one time, HBM factor c). However, for people with low HIV risk perception (HBM factor d) or low self-efficacy (HBM factor e), these characteristics may negate the benefit of reducing barriers and providing a cue to action. Those who do not feel that they are at high risk for HIV and those who do not feel able to self-administer an HIVST may be hesitant to test even, or especially, when HIVST is available. In addition, truckers have historically been characterized as having highly fatalistic views [28, 29], feeling that they have little control over their lives and health outcomes, which could prevent them from seeing the benefits associated with health promotion and disease prevention behaviors (HBM factor f). Thus, the benefits of adding HIVST in reducing barriers and serving as a cue to action to test may depend on other relevant psychosocial characteristics of the individual, such as HIV risk perception, self-efficacy, and fatalism. Therefore, the aim of this analysis was to assess if the impact of making HIVST available to truckers in Kenya on HIV testing over 6-month follow-up was modified by HIV risk perception, self-efficacy or fatalism.

## Materials and Methods

### Study Design and Data Collection

We used data from an RCT evaluating the impact of adding free oral HIVST as an HIV testing option for trucker clients of a network of eight roadside wellness clinics in Kenya run by the North Star Alliance. The trial has been described elsewhere [5, 25], but here we provide a brief description. Between October and December of 2015, all truckers who visited two of the clinics were invited to screen for eligibility to participate in a study on HIV testing. The eligibility criteria were: (a) 18 + years old, (b) male, (c) worked as a truck driver, (d) resided in Kenya, (e) spoke English or Kiswahili, (f) self-reported being HIV-negative or of unknown HIV status, (g) were able to sign the informed consent form, and (h) were willing to receive participation compensation via MPesa (a mobile phone-based money transfer system). The study enrolled 305 truck drivers, all of whom completed a baseline questionnaire, which included questions about demographics, sexual behavior and work, and various psychosocial scales. After the baseline questionnaire, participants were randomized on a 1:1 ratio, stratified on recruitment clinic, to the intervention arm (*n* = 150) or standard of care arm (SOC, *n* = 155). To avoid selection bias and crossover, participants were not informed of the exact study question nor that they would be randomized to be offered different HIV-testing options (masking).

Participants randomized to the SOC arm were offered SOC HIV testing with a rapid provider-administered finger-prick test in the clinic, as is standard protocol (all clients who come to the clinics for any reason are offered HIV testing in addition to any other services they need), and standard pre- and post-test counseling. Those randomized to the intervention arm were given a demonstration of the rapid oral HIVST and then offered a choice between the SOC or the HIVST while still in the clinic and were informed that they could pick-up HIVST kits from any of the eight clinics in Kenya over the following 6 months for home use or use in the clinic with supervision, depending on their preference. At 6-month follow-up, participants were interviewed and asked about any HIV testing over the past 6-months, whether through the North Star Alliance clinics (self-testing or SOC testing) or elsewhere. Data were collected by the interviewer on paper forms, which were transported to the main office and entered into a REDCap database for cleaning and analysis. Participants were compensated for their time with the equivalent of $6 US for completing the baseline and $4 US for completing the 6-month interview. At 6-month follow-up, 21 participants could not be contacted and were deemed lost to follow-up (8/150 in the intervention arm and 13/155 in the SOC arm), yielding a sample of 284 participants for these analyses [25].

The study was approved by the City University of New York (CUNY) Institutional Review Board, the Kenya Medical Research Institute (KEMRI) Ethics Committee, and the University of KwaZulu-Natal Biomedical Research Ethics Committee (BREC). All study participants provided signed informed consent.

### Measures

The exposure was an indicator for intervention group (versus the SOC). The outcome was any HIV testing over the 6-month follow-up period with either HIVST or SOC testing. The effect modifiers were:

1. *HIV risk perception* was assessed by asking: “Thinking about your situation now, what do you think are your chances of getting infected with HIV? Would you say no risk at all, a small risk, a moderate risk, or a great risk?” We recoded responses into an indicator for high HIV risk (great or moderate risk) versus low HIV risk (no or small risk).
2. *General self-efficacy* was assessed using a 10-item scale that had been validated in Europe and Asia [30]. Questions asked about the participant’s confidence that he could cope with a range of challenging demands. Response options were on a four-point Likert scale ranging from “not at all true” to “exactly true,” with higher scores indicating higher self-efficacy. The scale demonstrated excellent reliability in our sample (Cronbach’s α = 0.89) [31].
3. *Fatalism* was assessed using a 20-item scale [32] that elicited responses to a series of statements related to predetermination (e.g., “How long I live is predetermined”), luck (e.g., “I will get diseases if I am unlucky”), and pessimism (e.g., “Everything that can go wrong for me does”). Response options were on a 5-point Likert scale ranging from “strongly disagree” to “strongly agree,” with higher scores indicating more fatalistic views. The scale demonstrated excellent reliability in our sample (Cronbach’s α = 0.93).

Characteristics of the study participants collected at baseline were described for those who completed follow-up. Participant age ranged from 21 to 62 years and was examined in three categories (21–30, 31–40, and 41–62 years). Education was dichotomized into an indicator for high school graduate versus less than a high school degree. Marital status was dichotomized into an indicator for married (legally, common-law, or living with a partner) versus unmarried. Sexual risk behaviors examined included indicators for having > 1 partner, having paid for sex in the past 6 months, having had condomless sex in the past 6 months, and having a regular partner along the trucking route in addition to a wife or girlfriend at home during the past 6 months. Work-related factors included: travelling alone (versus with an assistant or family member), spending > 24 of the past 30 nights away from home for work, and trucking income (8,000–23,999 Kenyan shillings [KES] versus 24,000–55,000 KES per month, equivalent to approximately $80-$234 US versus $235-$540 US at the time of the trial).

### Statistical Analysis

We described the sample that completed follow-up overall and by HIV testing over follow-up, assessing the statistical significance of differences using the Pearson’s chi square test (Fisher’s exact when expected cell counts were < 5) for categorical variables and the Wilcoxon rank sum test for numeric variables. We then ran univariate log binomial regression to assess the association (risk ratio [RR]) of the intervention and each effect modifier with HIV testing over follow-up. We tested each effect modifier in a separate model by adding the effect modifier and the effect modifier*intervention interaction term to the model. All models that included the intervention were adjusted for recruitment clinic since randomization was stratified on clinic. We did not adjust models for other variables due to the randomization, which should prevent confounding. If the interaction term was significant, we ran the model stratified on the effect modifier to determine the direction of the effect modification. For significant numeric effect modifiers, we stratified on the median score.

We also assessed effect modification on the additive scale, which may be of more public health relevance than multiplicative interaction [33], calculating risk differences and testing for additive interaction using linear binomial regression [34]. If statistically significant, we reported risk differences (RD) stratified on the effect modifiers.

Although one participant in the intervention arm was not offered HIVST, all analyses were based on intent-to-treat because in our previous analyses there was no difference in results between the intent-to-treat and per protocol analyses [25]. Analyses were conducted using SAS 3.8 Enterprise Edition (Cary, NC) and significance was set at α = 0.05.

## Results

### Description of the Sample

Half (49.3%) of the participants were aged 31–40 with the other half almost evenly distributed between 21 and 30 (23.9%) and 41–62 (26.8%) years. Thirty-seven percent had completed high school and 83% were married or co-habiting. Sexual risk behavior reported at baseline was high, with 57.1% having had more than one partner in the past six months, 55.3% having paid for sex in the past 6 months, and 54.6% reporting a regular partner along the trucking route in addition to a wife or girlfriend at home. Eighty-seven percent reported condomless sex in the past 6 months. When examining work-related factors, nearly three-quarters (73.2%) of participants were in the lower income category (8,000–23,999 KES). About 41.9% traveled alone and 32.1% had spent more than 24 of the past 30 nights away from home for work. About half (51.6%) of participants felt that they had no or low risk of HIV infection. The mean score on the self-efficacy measure was 36.3 (possible score range 10–40), and 46.9 on the fatalism measure (possible score range 20–100) (Table 1).

**Table 1:** Description of the sample overall and stratified by HIV testing over the 6-month follow-up period

Variable	Total	Tested for HIV during follow up		χ2 (*P*-Value)
		Tested	Not Tested	
	N (%)	N (%)	N (%)	
**Total**	284 (100)	159 (56.0)	125 (44.0)	NA
**Intervention arm**				0.01 (0.905)
**Choice**	142 (50)	80 (56.3)	62 (43.7)	
**SOC**	142 (50)	79 (55.6)	63 (44.4)	
**Clinic location**				0.97 (0.324)
**Salgaa**	157 (55.3)	92 (58.6)	65 (41.4)	
**Maai Mahui**	127 (44.7)	67 (52.8)	60 (47.2)	
**Age (years)**				2.17 (0.337)
**21–30**	68 (23.9)	36 (52.9)	32 (47.1)	
**31–40**	140 (49.3)	75 (53.6)	65 (46.4)	
**41–62**	76 (26.8)	48 (63.2)	28 (36.8)	
**High School Graduate**				**5.21 (0.023)**
**Yes**	105 (37.0)	68 (64.8)	37 (35.2)	
**No**	179 (63.0)	91 (50.8)	88 (49.2)	
**Married (legal or common law/living with partner)**				0.001 (0.973)
**Yes**	234 (83.0)	131 (56.0)	103 (44.0)	
**No**	48 (17.0)	27 (56.3)	21 (43.8)	
**Sexual Risk Behavior**				
**Had > 1 partner in the past 6 months**				0.68 (0.412)
**Yes**	157 (57.1)	84 (53.5)	73 (46.5)	
**No**	118 (42.9)	69 (58.5)	49 (41.5)	
**Paid for sex in the past 6 months**				1.41 (0.235)
**Yes**	147 (55.3)	77 (52.4)	70 (47.6)	
**No**	119 (44.7)	71 (59.7)	48 (40.3)	
**Having a regular partner along the trucking route in addition to wife/girlfriend**				1.03 (0.311)
**Yes**	129 (45.4)	68 (52.7)	61 (47.3)	
**No**	155 (54.6)	91 (58.7)	64 (41.3)	
**Condomless sex in the past 6 months**				0.27 (0.603)
**Yes**	235 (87.0)	130 (55.3)	105 (44.7)	
**No**	35 (13.0)	21 (60.0)	14 (40.0)	
**Work-related factors**				
**Income per month from truck driver job**				0.07 (0.797)
**8**,**000–23**,**999 KES**	194 (73.2)	110 (56.7)	84 (43.3)	
**24**,**000–55**,**000 KES**	71 (26.8)	39 (54.9)	32 (45.1)	
**Travel alone (i.e. no an assistant or family member travels with participants during work)**				0.40 (0.525)
**Yes**	119 (41.9)	64 (53.8)	55 (46.2)	
**No**	165 (58.1)	95 (57.6)	70 (42.4)	
**Spent > 24 of the past 30 nights away from home for work**				0.002 (0.967)
**Yes**	89 (32.1)	49 (55.1)	40 (44.9)	
**No**	188 (67.9)	104 (55.3)	84 (44.7)	
**Psychosocial measures**				
**HIV Risk Perception**				0.22 (0.636)
**Low Risk**	146 (51.6)	84 (57.5)	62 (42.5)	
**High Risk**	137 (48.4)	75 (54.7)	62 (45.3)	
**Self-Efficacy Scale**				0.14 (0.889)*
**Mean (SD)**	36.3 (4.9)	36.7 (4.2)	36.4 (5.2)	
**Median (Range)**	38.0 (22.0–40.0)	38.0 (25.0–40.0)	39.0 (22.0–40.0)	
**Fatalism**				0.51 (0.611)*
**Mean (SD)**	46.9 (20.7)	45.7 (20.1)	47.2 (21.8)	
**Median (Range)**	48.0 (20.0–97.0)	45.0 (20.0–92.0)	49.0 (20.0–97.0)	

* Wilcoxon Rank Sum test statistic (*p*-value)

Overall, 159 people (56.0%) tested for HIV during the six-month follow-up period. The only characteristic significantly associated with testing was education, with high school graduates (64.8%) more likely to test over follow-up than those who had not completed high school (50.8%, *P* = 0.023) (Table 1).

### Regression Models Results

In the regression model adjusted only for clinic, there was no significant difference in HIV testing over follow-up by intervention arm on the multiplicative or the additive scales (RR = 1.0, 95% Confidence Interval [CI]: 0.8, 1.2, RD = 0.8 per 100, 95% CI: -10.7, 12.3). The three psychosocial measures also were not significantly associated with HIV testing over follow-up (Table 2).

**Table 2:** Association of adding HIV self-testing to an HIV testing program for truckers over 6 month follow-up and assessment of interaction with HIV risk perception, self-efficacy and fatalism

Variable		Univariate Models				Model with intervention, one effect modifier and the interaction term^a^
	Sample Size	Risk Ratio (95% CI)	Wald χ2 (*P*-value)	Risk Difference per 100 (95% CI)	Wald χ2 (*P*-value)	Wald χ2 (*P*-value) for interaction term
**Intervention (versus SOC)** ^**a**^	284	1.0 (0.8,1.2)	0.01 (0.924)	0.84 (-10.66, 12.32)	0.02 (0.887)	NA
**Psychosocial measures**						
**High HIV risk perception**	283	1.0 (0.8, 1.2)	0.06 (0.809)	-2.1 (-13.7, 9.5)	0.12 (0.727)	0.22 (0.642)^b^ 0.11 (0.735)^c^
**Self-Efficacy scale (higher score = higher self-efficacy)**	284	1.0 (1.0, 1.0)	0.10 (0.756)	0.3 (-1.0, 1.5)	0.15 (0.695)	0.51 (0.474)^b^ 0.47 (0.492)^c^
**Fatalism scale (higher score = more fatalistic views)**	284	1.0 (1.0, 1.0)	0.93 (0.335)	-0.2 (-0.4, 0.1)	1.02 (0.313)	**4.82 (0.030)** ^b^ **4.83 (0.030)** ^c^

^**a**^Adjusted for clinic of recruitment due to stratified randomization

^b^ Multiplicative scale

^c^ Additive scale

### Effect Modification Analysis

Significant interaction was observed between the intervention and fatalism on both the multiplicative (*P* = 0.030) and the additive (*P* = 0.030) scales (Table 2). In the stratified models, among those with low fatalism, the intervention was associated with higher HIV testing probability (RR = 1.3, CI: 1.0, 1.7, RD = 14.0 per 100, CI: -1.7, 29.6) whereas among those with high fatalism, those in the intervention arm had a lower probability of HIV testing compared to those in the SOC (RR = 0.7, CI: 0.5, 1.0; RD=-14.7 per 100, CI: -31.4, 2.1). All *p*-values were of borderline statistical significance (< 0.1) (Table 3).

**Table 3:** Association of intervention with HIV-testing over 6-month follow-up stratified on Fatalism

	Risk Ratio (95% CI)	Wald χ2 (*P*-value)	Risk Difference per 100 (95% CI)	Z (*P*-value)
Intervention impact among those with low fatalism^a^ (*n* = 150)	1.3 (1.0, 1.7)	3.40 (0.065)	14.0 (-1.7, 29.6)	1.75 (0.080)
Intervention impact among those with high fatalism^a^ (*n* = 134)	0.7 (0.5, 1.0)	3.24 (0.072)	-14.7 (-31.4, 2.1)	-1.72 (0.086)

^a^Fatalism was dichotomized at the median score of 48

The interactions for HIV risk perception and self-efficacy were not significant on the multiplicative scale (*P* = 0.642 and *P* = 0.474, respectively) or the additive scale (*P* = 0.735 and *P* = 0.492, respectively) (Table 2).

## Discussion

Previous analyses found that HIV testing was higher among those in the HIVST intervention than the SOC at baseline [5], but there was no difference over 6-month follow-up [25]. In this paper we also found HIV risk perception, self-efficacy, and fatalism were not significantly associated with HIV testing over 6-month follow-up. This is similar to a study in West Africa that found fatalism scores were not associated with HIV testing [35]. However, when we explored whether the HIVST intervention had an impact over follow-up in certain subgroups based on the HBM [26, 27], we found significant effect modification by fatalism on both the multiplicative and the additive scales such that the intervention was associated with an increase in HIV testing among those with low fatalism but a decrease among those with high fatalism, which explains our null findings when looking at the main effect of the intervention in the sample as a whole. This finding is consistent with the HBM, as those with highly fatalistic views may not perceive the benefits of HIV testing and this may negate the reduced barriers and/or cue to action associated with making HIVST available to this high-risk population. The literature suggests three types of fatalism that result in different reactions to a cue to action, or nudge, as the authors refer to it. One type is “passive” fatalism, in which the world is seen as beyond one’s control. Passive fatalists are unlikely to react even when a cue to action is presented. The other types of fatalism are “protective” and “pathological” and people with these types of fatalism are likely to react to a cue to action, although not always in positive ways [36]. Our findings are consistent with what might be expected from “passive” fatalism. Interventions that reduce barriers to testing and/or serve as a cue to action, such as offering a new biomedical technology for free, may be insufficient to encourage HIV testing among truckers with fatalistic views. One potential way to address passive fatalism is to make HIV testing a default (i.e. make HIV testing the default and people can refuse it instead of asking if they want HIV testing) [36]. This might explain why truckers were more likely to test at baseline when offered testing choices in the clinic as the questions changed from the SOC “would you like an HIV test?” to “which HIV test would you like?” However, choices might be difficult with HIVST for home use. Although providers can give people HIVST kits as the default rather than asking if they want a HIVST kit, it seems unlikely that passive fatalistic people will use the test kit they are given. Instead, interventions to address the fatalism that is common among truckers may be needed to increase regular HIV testing in this high-risk group. Fatalism associated with certain occupations may be attributed to institutional isolation and powerlessness [36]. Truckers may feel that their work involves impossible demands and lack of resources, and they feel powerless to improve conditions. The trucking industry may need to acknowledge these challenges and the impact they have on the health of their workers and explore how to address the problem.

This study had several limitations. First, all measures other than the intervention were based on self-report, and there may have been social desirability bias in reporting HIV testing over follow-up or misunderstanding of the questions asked in the psychosocial measures, leading to measurement error. In addition, because of the moderate sample size for these analyses, the lack of significant associations could be type 2 errors, especially regarding the assessment of effect modification, which requires a larger sample than assessment of main effects. Although the intervention was assigned randomly, which minimized the likelihood of confounding, confounding cannot be completely ruled out. While loss to follow-up could have led to selection bias, there was little difference by intervention arm (5% in the intervention arm versus 8% in the SOC, *P* = 0.292) and would therefore likely bias the results toward the null. However, we did rerun the analyses adjusting for potential confounders (age, education, marital status, income, HIV risk behaviors) and the results were similar. Since study participants were recruited from two North Star Alliance roadside wellness clinics in Kenya, results cannot be generalized to other truckers in Kenya, especially those who do not access healthcare, nor truckers in other countries or people working in other professions. Finally, this trial was completed in 2015 and HIVST is now approved for use in Kenya. The increasing accessibility of and familiarity with HIVST might lead to different associations if the same study were to be conducted today.

## Conclusions

We found that adding HIVST as a testing option increased HIV test uptake at baseline but not over 6-month follow-up overall. However, the intervention was effective in increasing 6-month HIV testing among truckers with low fatalism. Few studies have reported on the long-term impact of making HIVST available and none have evaluated fatalism, a common trait among truckers, as an effect modifier. Further research is needed to identify the subgroups for whom making HIVST available can have the greatest impact and to develop alternate interventions targeting those still not accessing testing even when HIVST is made available.

## Data Availability

The RCT protocol was registered prior to study commencement at the Registry for International Impact Evaluations (RIDIE-STUDY-ID-55847d64a454f): https://ridie.3ieimpact.org/index.php?r=search/detailView&id=342. The data from this study are publicly available in the Harvard Dataverse repository: https://dataverse.harvard.edu/dataset.xhtml? persistentId=doi:10.7910/DVN/8GVXJY.
